# Journal Production Guidance for Software and Data Citations

**DOI:** 10.1038/s41597-023-02491-7

**Published:** 2023-09-26

**Authors:** Shelley Stall, Geoffrey Bilder, Matthew Cannon, Neil Chue Hong, Scott Edmunds, Christopher C. Erdmann, Michael Evans, Rosemary Farmer, Patricia Feeney, Michael Friedman, Matthew Giampoala, R. Brooks Hanson, Melissa Harrison, Dimitris Karaiskos, Daniel S. Katz, Viviana Letizia, Vincent Lizzi, Catriona MacCallum, August Muench, Kate Perry, Howard Ratner, Uwe Schindler, Brian Sedora, Martina Stockhause, Randy Townsend, Jake Yeston, Timothy Clark

**Affiliations:** 1 American Geophysical Union, 2000 Florida Ave. NW, Washington, DC 20009 USA; 2 Crossref, Lynnfield, MA 01940 USA; 3 Taylor & Francis, London, UK; 4 SSI/University of Edinburgh, Edinburgh, UK; 5 GigaScience Press, New Territories, Hong Kong SAR; 6 Michael J. Fox Foundation, New York, NY 10163 USA; 7 F1000Research, London, UK; 8 Wiley, Hoboken, NJ 07030 USA; 9 American Meteorological Society, Boston, MA 02108 USA; 10 EMBL-EBI, Cambridgeshire, UK; 11 Atypon, 111 River Street, Hoboken, NJ 07030 USA; 12 University of Illinois at Urbana-Champaign, Urbana, IL 61801 USA; 13 Elsevier, Radarweg 29, 1043 NX Amsterdam, The Netherlands; 14 Hindawi, 1 Fitzroy Square, London, W1T 5HF UK; 15 American Astronomical Society, Washington, DC 20006 USA; 16 CHORUS, 72 Dreyer Avenue, Staten Island, NY 10314 USA; 17 PANGAEA, Bremen, Germany; 18 DKRZ/IPCC DDC German Climate Computing Center (DKRZ), Hamburg, Germany; 19 Plos, 1265 Battery Street, San Francisco, CA 94111 USA; 20 AAAS, 1200 New York Ave NW, Washington, DC 20005 USA; 21 University of Virginia, Charlottesville, VA 22904 USA

**Keywords:** Publication characteristics, Media formats

## Abstract

Software and data citation are emerging best practices in scholarly communication. This article provides structured guidance to the academic publishing community on how to implement software and data citation in publishing workflows. These best practices support the verifiability and reproducibility of academic and scientific results, sharing and reuse of valuable data and software tools, and attribution to the creators of the software and data. While data citation is increasingly well-established, software citation is rapidly maturing. Software is now recognized as a key research result and resource, requiring the same level of transparency, accessibility, and disclosure as data. Software and data that support academic or scientific results should be preserved and shared in scientific repositories that support these digital object types for discovery, transparency, and use by other researchers. These goals can be supported by citing these products in the Reference Section of articles and effectively associating them to the software and data preserved in scientific repositories. Publishers need to markup these references in a specific way to enable downstream processes.

## Introduction

Software and data citation are emerging best practices in academic and scientific communication that provide excellent support for results validation, reproducibility, credit, sharing and reuse of valuable tools and resources. Data citation has been possible with some rigor since the establishment of DataCite—a Registration Agency of the International DOI Foundation, which issues robust persistent identifiers for datasets and maintains a registry of associated rich metadata—in 2009^1^ and was recommended by a comprehensive report of a CODATA-ICSTI task group in 2012 (available at: http://www.nap.edu/catalog.php?record_id=13564). CODATA-ICSTI is the international and interdisciplinary Committee on Data for Science and Technology (CODATA) and the International Council for Scientific and Technical Information (ICSTI) jointly-formed Task Group on Data Citation Standards and Practices (https://codata.org/initiatives/task-groups/previous-tgs/data-citation-standards-and-practices/). It has become increasingly adopted since the introduction of the “Joint Declaration of Data Citation Principles”^2^ in 2014 and its endorsement by 125 publishers, academic institutions, and funders (see https://force11.org/info/endorse-the-data-citation-principles/). The enthusiasm for these principles prompted an effort to provide a clear set of practical guidelines for publishers to begin to implement data citations. In 2018 “A data citation roadmap for scientific publishers”^3^ was published, incorporating work from several groups via workshops and including major participation by representatives of Elsevier, Springer-Nature, PLOS, eLIFE, JISC, EMBO Press, CrossRef, and Wiley. However, even when following this guidance, several publishers were finding that machine-actionable software and data citations from their own published articles were not preserved intact when the article was published, and that downstream services and linking were not enabled. Furthermore, it should be noted that software usage and citation, while similar to data citation, has certain key differences that should be reflected in publication workflows^4,5^. These are complex and involve authors, reviewers, editors, and other infrastructure services. Here we provide updates on this guidance to enable automated attribution and credit for software and data used in published articles.

Funders and advisory groups are now beginning to require data and software citation in order to connect publications with their supporting software and data(see, for example, https://wellcome.org/grant-funding/guidance/data-software-materials-management-and-sharing-policy). Notably, the Intergovernmental Panel on Climate Change (IPCC) enhanced the traceability of its recent Assessment by the implementation of the FAIR (Findable, Accessible, Interoperable, and Reusable) Guidelines^6,7^, which extends IPCC’s Assessment process by the documentation, long-term preservation, and citation of the assessed digital information, including both data and software. Recently, the White House Office of Science and Technology (OSTP) Memorandum “Ensuring Free, Immediate, and Equitable Access to Federally Funded Research”^8^ has directed federal agencies to develop implementation plans around open access for research results attributed to grants, including to the software and data.

Recommended software citation practices have more recently been clarified by the FORCE11 Software Citation Implementation Working Group in “Recognizing the value of software: a software citation guide”^5^. FORCE11 is a community of scholars, librarians, archivists, publishers and research funders that has arisen organically to help facilitate the change toward improved knowledge creation and sharing (https://force11.org/info/about-force11/). With the current intensive use of software including specialized tools and models for scientific research problems, software has evolved to become a key research resource requiring transparency and disclosure to the readers of any academic or scientific article. With work by the FORCE11 Software Citation Implementation Working Group, citation of software can now be consistently implemented by publishers who follow the guidelines in this article, establishing both data citation and software citation as important elements in peer-reviewed articles.

Long-term preservation and sharing of software and data that support research results in trustworthy scientific repositories, preferably domain-specific repositories^9^, provide for discovery, transparency, validation, and re-use by other researchers. Publishers should provide the means to associate these products to research articles through citation in the references section, using the structured implementation guidance for software and data citation in publishing workflows, provided in this article. Software citation, like data citation, provides a direct path to FAIRness for these essential research components^10,11^. The updated guidance provided here includes recent improvements in the practices, challenges publishers are encountering, and recommendations for addressing those challenges.

## Results

Journal publishers should make best efforts to undertake the following practices, on which we provide further detailed guidance in this article.

**Software and Data Citations: Checklist of Best Practices for Journal Publishers**

1. **Instructions to Authors:** Provide author instructions that define which software and datasets should be cited in their article, reinforce these instructions throughout the process including editorial actions. Additionally, include how to structure these citations and provide information on selecting the best possible scientific repositories to use for software and data, and what information to put in an Availability Statement.
2. **Publication Policies:** Update publication policies to include the importance of citing the software and datasets that support the transparency, integrity, and reproducibility of the research.
3. **Education and Outreach:** Educate editors, reviewers, and staff on the policy, requirements, areas of flexibility, and unique aspects of software and dataset citations.
4. **Technical Updates:** Put into place the necessary technical updates, as defined in this document, to ensure that the machine-actionable representation of the software and dataset citations is sustained throughout the publication workflow and properly formatted when registered to Crossref.
5. **Production Teams:** Define for publication production team members the unique aspects of software and dataset citations; work together to implement necessary changes.
6. **Metrics:** Establish quality metrics and measures to ensure consistent, accurate results for software and dataset citations.

## Discussion

This document is the product of working sessions of the FORCE11 Software Citation Implementation Working Group’s Journals Task Force, conducted over a period of 2 years. It reflects lessons learned from a project supported by an NSF Grant (2025364) to the American Geophysical Union to ensure that software and data citations were transferred reliably from publications to NSF’s Public Access Repository. It provides best practices to journals such that the machine-actionable representation of the software and dataset citations is sustained throughout the publication workflow and properly formatted when deposited to Crossref. This optimizes their ability to be properly linked as research objects in support of services provided by DataCite, Scholarly Link eXchange (Scholix), and others. The FORCE11 Journals Task Force includes both society and commercial publishers of all sizes, research infrastructure providers, and others interested in implementation challenges for software and data citations.

The guidance here is primarily intended for those who support the journal production process, including those engaged in copy editing, markup, and preparing the article for publication on various types of digital and print platforms. To implement these recommendations, coordination with journal submission systems, author guidelines, editors, reviewers, authors, and others involved with the front-end process of publications will also need to be included. Since journal production depends on a number of activities that take place both prior and subsequent to production in the publication workflow, we include brief descriptions of what is expected in those activities.

In this guide we describe a set of necessary journal-related activities, organized by role, along with what is needed for datasets and software that supports the article to be identified and cited. We provide use cases for journals to consider as they ensure that their production processes are handling data and software citations optimally.

### Problem statement

Data and software citations have unique use cases and workflows that distinguish them from article or book citations. In particular, software and data are often registered by Digital Object Identifier (DOI) registration agencies other than Crossref.

We have discovered that at many publishers, when scripts in the journal production process attempt to validate these citations, they do not do so correctly, and as a result, the integrity of the machine-actionable link to the software or dataset is not maintained and not provided to Crossref. As a result, important downstream services are not supported.

The global research community is now advancing the importance of citing software and datasets in scholarly literature as a means to be more transparent and support steps towards better reproducibility and reusability of software and datasets. Data and software are research objects separate from the article with the potential of reuse and citation in later works. Tracking these citations accurately so that authors/creators of these objects receive recognition is important and ensures the scholarly record is more complete.

Journal practices are influenced by other participants in the research ecosystem and the processes and services they provide. For this set of recommendations, these include Crossref (or other DOI registration agencies), Event Data (a joint service by Crossref and DataCite), DataCite Commons, and Scholix. These work together to associate software and datasets with publications for many other uses.

Crossref provides DOI registration services for publishers of English language peer-reviewed articles, their journals, and other publications. Using the Reference Section of each registered article, Event Data (a joint service by Crossref and DataCite that captures online mentions of Crossref records, https://www.crossref.org/blog/event-data-now-with-added-references/) records the relationship between the software and datasets cited in the article with the article itself in a database that other services can use. Event Data can also be populated if a relationship is asserted (with an identifier) to software or datasets in the journal metadata. Crossref has encountered challenges in providing consistent support to Event Data. In 2021, Crossref completed updates that fixed and improved these processes; however, incorrect publisher markup of the software and dataset citations still prevents full implementation.

DataCite, like Crossref, provides DOI registration services. DataCite specializes in preserving scientific datasets, some preprint servers (e.g., arXiv.org), physical samples (e.g., International Generic Sample Numbers (IGSNs),), and software (e.g., Zenodo). DataCite is the preferred DOI registration agency for datasets and software because of its robust identifier system, metadata, and support services.

DataCite Commons uses Event Data as well as the metadata from both Crossref and DataCite’s APIs (Application Programming Interface), ORCIDs (Open Researcher and Contributor ID, https://orcid.org/), and RORs (Research Organization Registry, https://ror.org/) to visualize the connections between works (e.g., article, data, software), people, organizations, and repositories. For example, you can see the works that cite a particular dataset.

Scholix is an interoperability framework that collects and exchanges links between research data and literature^12,13^. Scholix is designed to support multiple types of persistent identifiers, although at the moment only DOIs are included. Both Crossref and DataCite feed DOIs to Scholix. If a data repository includes information about related publications that use a dataset, through the metadata included in the DOI registration process (as metadata property ‘relatedIdentifier’), DataCite will provide that information (linked to the newly created dataset DOI) to Scholix. Similarly, if an article includes a dataset in the Reference Section, Crossref will report that to Scholix. Scholix manages multiple entries when the same article-to-dataset link is provided by two different sources. Software is not supported at this time.

### Workflow description

The following activities describe a typical publishing workflow, grouped by role, used to capture software and dataset citations properly in both human-readable and machine-actionable formats to support linking and automated attribution and credit. The order of activities may be slightly different for each publisher’s production process.

**Author Preparation**

1. Use scientific, trustworthy repositories that register data and software with persistent, globally unique and resolvable identifiers. Trustworthy repositories are key to helping authors make their data and software as well-documented, accessible, and reusable as possible. These qualities are embodied in the FAIR Guiding Principles. Datasets and software that are as FAIR and openly licensed as possible should be encouraged by journals, taking into consideration necessary protections and national laws. Trusted repositories provide long-term curation and preservation data and software services guided by the TRUST (Transparency, Responsibility, User focus, Sustainability and Technology) principles^14^. CoreTrustSeal — an international, community based, non-governmental, and non-profit organization promoting sustainable and trustworthy data infrastructures, and offers to any interested data repository a core level certification based on the Core Trustworthy Data Repositories Requirements — provides a list of certified repositories that follow those principles^15^. Specific dataset curation services may require time to review and prepare the data prior to publication. This is most common with repositories that specialize in a particular scientific domain or data type. The time needed for domain-specific data curation, the value-added process of making the dataset interoperable and more likely to be reused, may be up to several weeks or more. Generalist repositories such as Harvard Dataverse Repository, Dryad, and Zenodo are typically far quicker as they do not undertake domain-specific curation. However, domain-specific repositories may be the norm for certain disciplines. Journals should encourage researchers to be aware of this time constraint where applicable, and to plan accordingly. Well-curated data provides transparency and integrity to research. The repository landscape is growing to accommodate the needs of researchers. Tools like re3data.org, DataCite Commons (using both DataCite and re3data.org content), FAIRsharing.org, or the CoreTrustSeal website are well-managed and support authors in finding a trustworthy discipline-specific repository.
  Software is best preserved in repositories that supports versioning. Researchers can use re3data.org to locate software preservation repositories. Those researchers that use GitHub.org as a development platform can use the integrated bridge to Zenodo.org to preserve a specific version for publication. When using the Zenodo bridge remind your authors to double-check author names, add in author ORCIDs, and make necessary corrections to citation information and metadata.
2. Include in the submitted article both an Availability Statement for software and datasets and citations in the Reference Section paired with in-text citations in the article. Publishers should provide support with author guidance and journal templates. All software and datasets can be described in the Availability Statement, but frequently the information needed for a complete citation is not fully available.
  - **An Availability Statement** is designed for the reader and states where the supporting datasets and software are located and any information about accessibility that is needed. Include an in-text citation that has a corresponding entry in the Reference Section. This is a statement on availability so that those looking to analyze or reuse datasets or software can easily find these objects. The information provided should lead the reader to the exact location where the software or dataset can be found. See Availability Statement Recommended Elements Section for more information.
  - **Paired Citations and References**
    i. **In-text citations** in the Methods and/or Data Section that refer to corresponding entries (citations) in the Reference Section for the dataset or software. These in-text citations provide discrete linkage from research claims to the specific dataset or software supporting them and/or methods used in analysis similar to citations of research articles or other sources.
    ii. **Citation (in Reference Section)** for the dataset and software used in this research. These should be listed in the Reference Section. This allows for automated attribution and credit through Crossref’s Event Data. See section Techniques for Identifying a Data or Software Citation in your References Section Metadata for a suggestion on using a “bracketed description” in the citation format to make identification of data and software citations easier. Also, see Software and Data Citation Resources Section.
3. **Be aware that reference tools used by many authors may not have an item type for datasets or software yet**. In order to follow the best practices in this guide, further editing of the citation may be required. For example, Zotero does not support datasets, but does support software. Zotero provides a workaround to support datasets.

**Journal staff review**

1. **Ensure the presence of a sufficiently descriptive Availability Statement with actionable DOI/URLs** for both software and datasets supporting the research.
2. **Ensure that for all software and datasets identified in the Availability Statement there is a supporting citation and the statement is complete**. Journal review guidance is helpful (e.g., American Geophysical Union’s (AGU) Data and Software Availability and Citation Checklist). The journal should promote the importance of a citation in the Reference Section as much as is feasible to encourage automated attribution and credit. Provide examples relevant to your journal. Citations should be to both newly created software and datasets as well as to software and datasets already deposited in repositories by other researchers and reused or referred to in the research by the authors. See Availability Statement Recommended Elements Section for more information.
3. Provide guidance to authors recommending that software and datasets in the Availability Statement be preserved for the long term in a trustworthy repository complying with the TRUST principles^14^ including its inherent long-term commitment for sustainable operations. DataCite has enhanced their Commons tool to include repositories; In addition, CoreTrustSeal provides a list of their certified repositories. Use keywords to discover potential repositories and review relevant characteristics like domain specialties, technical properties, governance, upload policies, and possible certifications^16^.
4. Ensure citation follows journal style guides. Journals use many different citation styles. Some styles are flexible enough to allow the author to assist with identifying the citation type by using a “bracketed description” to indicate the citation as a dataset, software, computational notebook, etc. This is important to inform automated and manual reference section review as well as accurate production markup. See Software and Data Citation Resources Section for more information.

**Reviewer/editor evaluation**

1. Review the submitted article to determine if the software and datasets referenced in the Methods and Data or Methods and Materials Section appears to fully support the research, such that all has been accounted for.
2. Review the Availability Statement to ensure all the software and datasets referenced are included. Use the information provided to locate and observe the software and datasets. Ensure that the links resolve and the content is as described. Request clarity as needed. Ensure the Availability Statement is complete. See Availability Statement Recommended Elements Section for more information.

**Publisher responsibility**

1. **Ensure the entire production process team, such as copy editors, markup vendors, and staff, are aware of the uniqueness of software and dataset citations**. This includes providing guidance, education, and support for questions. See the Software and Data Citation Resources Section for more information.
2. **Ensure the publication policies are current** using best practices of including software and dataset citations such as the Transparency and Openness Promotion Guidelines (TOP) Guidelines^17^ and the Research Data Policy Framework for All Journals and Publishers^18^ and those that extend this work to include software.
3. **Implement quality assurance processes** for measuring software and data citation accuracy.
4. **Establish quality controls, measures and metrics** to track consistency and establish thresholds for when to take action when the measures indicate a degradation in quality.

**Automated verification activity - reference section (Publisher or third-party vendor responsibility)**

1. **Check software and dataset citations in the Reference Section**. When checking citations, note that software and dataset citations formats can include repository names and version numbers. Guidance from FORCE11 and Research Data Alliance (RDA), a community-driven initiative by the European Commission, the United States Government’s National Science Foundation and National Institute of Standards and Technology, and the Australian Government’s Department of Innovation with the goal of building the social and technical infrastructure to enable open sharing and re-use of data (https://www.rd-alliance.org/about-rda) provides recommended formats. See the Software and Data Citation Resources Section for more information. The format for persistent identifiers for software and datasets can vary. DOIs are commonly registered through DataCite and potentially other DOI Registration Agencies. Consider using content negotiation to validate. Content Negotiation allows a user to request a particular representation of a web resource. DOI resolvers use content negotiation to provide different representations of metadata associated with DOIs. A content negotiated request to a DOI resolver is much like a standard HTTP request, except server-driven negotiation will take place based on the list of acceptable content types a client provides. (https://citation.crosscite.org/docs.html).
2. **Ensure that software and dataset citations are tagged accurately**. Avoid tagging them as “other”. Refer to JATS for Reuse (JATS4R) guidance for data citations^19^ and software citations from The National Information Standards Organization (NISO)^20^.
3. **Avoid removing citation information**, especially the persistent identifier.

**Copy editor review (re: language and style editing)**

**Check that the software and datasets mentioned in the Methods and Data Section have corresponding entries in the Availability Statement and citations in the Reference Section**.

Note: not all data or software can be supported with a citation that includes a persistent identifier.

**Production Markup Review (supports machine-actionable formats)**

1. **Methods/Data Section:** Check that in-text citations and/or text in the Availability Statement link correctly to citations.
2. **Availability Statement:** This text should include availability statements for all software and datasets that support the research. Currently most journals do not mark up this text. However, JATS4R now has a specific recommendation to do so.
3. **Citations:** Journals should review and update the markup requirements for dataset citation^19^ and software citation^20^. The persistent identifier or URL is an active link in the article to the reference. Some publishers provide visualization tools of the software or dataset. Consult the Software and Data Citation Resources Section of this article for information on formatting. Dataset and software research outputs should use the same journal citation style and treatment of the persistent identifier with slight adjustment to include software version information and bracketed descriptions.

**Content hosting provider activities**

1. **Register the article with Crossref and ensure metadata is sent to Crossref**, including the full reference list (and the Availability Statement when added to the Crossref schema). Ensure all citations are included in the file going to Crossref, and not being removed inadvertently. Use the Crossref Participation Report to check publisher metadata overview of what is provided to Crossref. Consult with Initiative for Open Citations (I4OC, https://i4oc.org/) for community recommendations on open citations.
2. **Use the Software and Dataset Citation Use Cases provided below as a guide to ensure coverage of most variations**.
3. **Display the human readable citation correctly**. Consult the Software and Data Citation Resources Section of this article for information on formatting. Dataset and software research outputs should use the same journal citation style and treatment of the persistent identifier with slight adjustment to include software version information and possibly bracketed descriptions.
4. **Provide the machine-actionable citation correctly** to downstream services (e.g., Crossref). It is important to note the guidance provided by Crossref in their blog (available at https://www.crossref.org/blog/how-do-you-deposit-data-citations/) has since been amended. If a journal implemented this guidance previously, there is a high probability that a correction is needed, using the information in this article. The Software and Dataset Citation Use Cases tables below (Tables 1–4), specifically the Crossref information, includes those corrections. In short, if there is a DOI, include the DOI XML tag.

**Table 1:** Software Citation Use Cases–Software Citation with registered DOI & Software Citation with URL (not persistent).

Use Case Name, Description	Desired Outcome for JATS XML version 1.3	Desired Outcome for Crossref Metadata depository schema 5.3.1	Notes
**Use case A** Software Citation with registered DOI A registered DOI has the desired metadata to support this citation and allows for content negotiation	<ref id=“bib11”> <element-citation publication-type=“software”> <person-group person-group-type=“author”> <name> <surname> Wang</surname> <given-names> Yafei </given-names> </name>…</person-group> <year iso-8601-date=“2022”> 2022 </year> <part-title> pc4covid19/pharmacodynamics2GUI: Version 2.0</part-title> <source> zenodo </source> <pub-id pub-id-type=“doi”> 10.5281/zenodo.3967327 </pub-id> </element-citation> </ref>	Tags: //citation/unstructured_citation AND //citation/doi e.g. <citation key=“XXX”> <unstructured_citation>Wang Yafei, …, 2022, pc4covid19//pharmacodynamics2GUI: Version 2.0, zenodo, 10.5281/zenodo.3967327</unstructured_citation> <doi> 10.5281/zenodo.3967327</doi> </citation>	Pro: Crossref can update Event Data. At this time software is not included in Scholix.
**Use Case B** Software Citation with URL (not persistent) e.g., GitHub, GitLab, BitBucket, The community accepted location for the software does not use persistent identifiers. A URL is provided instead. No DOI registration services are available.	<ref id=“bib3”> <element-citation publication-type=“software”> <person-group person-group-type=“author”> <name> <surname> Kotila </surname> <given-names> M </given-names> </name>…</person-group> <year iso-8601-date=“2019”>2019</year> <part-title>Numerical computing is fun</part-title> <source>GitHub</source> <ext-link ext-link-type=“uri” xlinkhref=“https://github.com/eka-foundation/numerical-computing-is-fun”>https://github.com/eka-foundation/numerical-computing-is-fun</ext-link> </element-citation> </ref>	Tags: //citation/unstructured_citation e.g. <citation key=“XXX”> <unstructured_citation> Kotila M, …, 2019, Numerical computing is fun, GitHub, https://github.com/eka-foundation/numerical-computing-is-fun” >https://github.com/eka-foundation/numerical-computing-is-fun </unstructured_citation> </citation>	Con: Software is not in a trusted preservation repository. The URL is subject to link rot and it is not FAIR.

**Table 2:** Software Citation Use Cases—Software Citation with Software Heritage Persistent Identifier (PID) & Software Citation with other PID.

Use Case Name, Description	Desired Outcome for JATS XML version 1.3	Desired Outcome for Crossref Metadata depository schema 5.3.1	Notes
**Use Case C** Software Citation with Software Heritage Persistent Identifier (PID) A registered PID has the desired metadata to support this citation and allows for content negotiation.	<ref id=“bib2”> <element-citation publication-type=“software”> <person-group person-group-type=“author”> <name> <surname> Zhang </surname> <given-names> Martin </given-names> </name> </person-group> <year iso-8601-date=“2021”>2021</year> <part-title> tabula-muris-senis </part-title> <source> Software Heritage </source> <pub-id pub-id-type=“swhid”> swh:1:dir:7dc782970300a97e9bca9038ba34728c857a0638 </pub-id> </element-citation> </ref>	Tag: //citation/unstructured_citation e.g. <citation key=“XXX”> <unstructured_citation> Zhang Martin 2021, tabula-muris-senis, Software Heritage,swh:1:dir:7dc782970300a97e9bca9038ba34728c857a0638 </unstructured_citation> </citation>	Con: Non-DOI PIDS are typically not converted to clickable links on journal websites and some journals encourage use of a URL as a preference.
**Use Case D** Software Citation with other PID (e.g., RRID (Research Resource Identification), ARKs (Archival Resource Key, https://registry.identifiers.org/registry/ark), Handle, ASCL (Astrophysics Source Code Library, https://registry.identifiers.org/registry/ascl), SwMathID), A PID is used for the software but is not a DOI.	<ref id=“bib2”> <element-citation publication-type=“software”> <person-group person-group-type=“author”> <collab> Broad Institute </collab> </person-group> <part-title> CellProfiler Image Analysis Software </part-title> <pub-id pub-id-type=“rrid”>SCR_007358</pub-id> </element-citation> </ref>	Tag: //citation/unstructured_citation e.g. <citation key=“XXX”> <unstructured_citation> Broad Institute, CellProfiler Image Analysis Software,SCR_007358 </unstructured_citation> </citation>	Con: Non-DOI PIDS are typically not converted to clickable links on journal websites.

**Table 3:** Data Citation Use Cases — Data Citation with registered DOI & Data Citation with unregistered DOI (DOI does not resolve).

Use Case Name, Description	Desired Outcome for JATS XML version 1.3	Desired Outcome for Crossref Metadata depository schema 5.3.1	Notes
**Use Case 1** Data Citation with registered DOI A registered DOI has the desired metadata to support this citation and allows for content negotiation.	Example: <ref id=“d1”> <element-citation publication-type=“data”> <person-group person-group-type=“author”>… <name> <surname> van Beethoven </surname> <given-names> Ludwig </given-names> </name>… </person-group> <data-title>Title of dataset </data-title> <year iso-8601-date=“2014”>2014 </year> <source> Repository Name </source> <pub-id pub-id-type=“doi” > 10.1234/1234321 </pub-id> </element-citation> </ref>	Tags: //citation/unstructured_citation AND //citation/doi e.g. <citation key=“XXX”> <unstructured_citation>van Beethoven Ludwig, 2014,Title of dataset, Repository Name, 10.1234/1234321 </unstructured_citation> <doi> 10.1234/1234321</doi> </citation> </citation>	Pro: Crossref can update Event Data and Scholix.
**Use Case 2** Data Citation with unregistered DOI (DOI does not resolve) Some data repositories do not complete the DOI registration process until after the article is published. The DOI is valid but will not resolve to a dataset yet. Likely a temporary “share link” was used for article peer review. Make sure the temporary link is not accidentally included here, instead of the persistent identifier.	Same as above	Tags: //citation/unstructured_citation e.g. <citation key=“XXX”> <unstructured_citation>van Beethoven Ludwig, 2014,Title of dataset, Repository Name, 10.1234/1234321</unstructured_citation> </citation>	Con: Crossref rejects the submission when a DOI tag is used. Only the “unstructured” tag can be used. The DOI is registered after the article is published, and cannot be used to validate the citation. Crossref can NOT update Event Data.

**Table 4:** Data Citation Use Cases — Data Citation with URL (not persistent) & Data Citation with non-DOI PID.

Use Case Name, Description	Desired Outcome for JATS XML version 1.3	Desired Outcome for Crossref Metadata depository schema 5.3.1	Notes
**Use Case 3** Data Citation with URL (not persistent) (e.g., project website, ftp site) The community accepted location for the data does not use persistent identifiers. A URL is provided instead. No DOI registration services are available.	Use <ext-link> to provide a link directly to the data citation. If there is no pub-id, ext-link should be included. <ref id=“bib11”> <element-citation publication-type=“data”> <person-group person-group-type=“author”> <name> <surname> Radoshevich </surname> <given-names>L</given-names> </name>… </person-group> <year iso-8601-date=“2015”> 2015 </year> <data-title> Title of dataset </data-title> <source> ArrayExpress </source> <ext-link ext-link-type=“uri” xlinkhref=“https://www.ebi.ac.uk/arrayexpress/experiments/E-MEXP-1243”>https://www.ebi.ac.uk/arrayexpress/experiments/E-MEXP-1243</ext-link> </element-citation> </ref>	Tags: //citation/unstructured_citation e.g. <citation key=“XXX”> <unstructured_citation>Radoshevich L, …,2015,Title of dataset, ArrayExpress, https://www.ebi.ac.uk/arrayexpress/experiments/E-MEXP-1243,</unstructured_citation > </citation>	Con: Data are not in a trusted preservation repository. Scholix is not updated. The URL is subject to link rot and it is not FAIR. Metadata about the data are difficult to determine.
**Use Case 4** Data Citation with non-DOI PID (e.g., RRID, ARKs. Handle, Accession) Additional information in the Software and Data Citation Resources section below. A PID is used for the data but is not a DOI.	<ref id=“bib11”> <element-citation publication-type=“data”> <person-group person-group-type=“author”> <name> <surname> Radoshevich </surname> <given-names> L </given-names> </name>… </person-group> <year iso-8601-date=“2015”>2015</year> <data-title> Title of dataset </data-title> <source> ArrayExpress</source> <pub-id pub-id-type=“accession” xlinkhref=“https://www.ebi.ac.uk/arrayexpress/experiments/E-MTAB-3649/“ >E-MTAB-3649</pub-id> </element-citation></ref>	Tags: //citation/unstructured_citation e.g.<citation key=“XXX”> <unstructured_citation>Radoshevich L, …,2015,Title of dataset, ArrayExpress,E-MTAB-3649, https://www.ebi.ac.uk/arrayexpress/experiments/E-MTAB-3649/</unstructured_citation> </citation>	Con: Non-DOI PIDs are not included in Scholix. Non-DOI PIDS are typically not converted to clickable links on journal websites and some journals encourage use of a URL as a preference.

**Crossref activities**

1. Receive the article XML from the **Content Hosting Provider/Publisher** and create the necessary Event Data entries for software and dataset citations included in the Reference Section.

We include below a list of specific software and dataset use cases with recommended JATS XML and Crossref submission record elements. JATS XML refers to Journal Article Tag Suite (JATS) Library, a standard publishers use to mark up journal article content in XML format. (https://www.niso.org/standards-committees/jats)

Following the use cases are recommendations for techniques for identifying a software or dataset citation in the Reference Section.

### Software and dataset citation use cases

Tables 1–4 list the common use cases for software (Tables 1, 2) and dataset (Tables 3, 4) citations, the corresponding JATS XML elements, and Crossref Metadata depository schema elements to assist publishers with creating the necessary adjustments to their production systems that result in proper machine-readable and actionable citations. The first use case for software citation (use case A in Table 1) and dataset citation (use case 1 in Table 3) represent the most desirable condition to support automated attribution and credit. Use cases not included are listed in text below. In each table, we show the desired outcome for JATS XML version 1.3, which is available at https://www.niso.org/publications/z3996-2021-jats. The example used is <element-citation> but <mixed-citation> is equally acceptable. We also show the desired outcome for Crossref Metadata depository schema 5.3.1, which is available at https://www.crossref.org/documentation/schema-library/metadata-deposit-schema-5-3-1/.

**Use cases not included in this guidance:**

1. Citation to a physical location such as a museum.
2. Mixed content citation that includes, for example, software, data, and other digital objects where individual attribution to a specific item is difficult or not possible.
3. Physical samples using IGSN, RRID, or other persistent identifiers.
4. Executable environments, for example, MyBinder, Executable Research Article (ERA)^21^, and Code Ocean.
5. Datasets associated with different persistent identifiers, for example journal supplemental files for a journal article where the journal article DOI is used for citation.
6. Datasets and software together with an online software implementation associated with a publication are reviewed, published and preserved long-term, used data and software is cited and the provenance documented: IPCC AtlasChapter with WGI Interactive Atlas and WGI Github Repository.

### Software and Data Citation - Crossref Metadata Schema examples

#### Software Citation Example

**Published Article**

Shumate A and Salzberg S. LiftoffTools: a toolkit for comparing gene annotations mapped between genome assemblies [version 1; peer review: 1 approved with reservations]. *F1000Research* 2022, 11:1230 (10.12688/f1000research.124059.1)

**Availability Statement (located in the ‘Software Availability’ Section in this article)** Archived source code as at time of publication: 10.5281/zenodo.6967163 (Shumate, 2022)

**Software Citation in Reference Section**

Shumate, A. (2022). agshumate/LiftoffTools: (v0.4.3.2) [Computer software]. Zenodo. 10.5281/ZENODO.6967163

**Crossref Metadata**


{"key": "ref6","doi-asserted-by": "publisher","unstructured": "Alaina Shumate, 2022, agshumate\/LiftoffTools: Version 0.4.3.2, zenodo, 10.5281\/zenodo.6967163","DOI": "10.5281\/zenodo.6967163"},


#### Data citation example

**Published Article**

Zhang, Y., Li, X., Liu, Z., Rong, X., Li, J., Zhou, Y., & Chen, S. (2022). Resolution Sensitivity of the GRIST Nonhydrostatic Model From 120 to 5 km (3.75 km) During the DYAMOND Winter. In Earth and Space Science (Vol. 9, Issue 9). American Geophysical Union (AGU). 10.1029/2022EA002401.

**Availability Statement (located in the Open Research Section in this article)**

Data for supporting this study are available at: https://zenodo.org/record/6497071 (GRIST-Dev, 2022).

**Data Citation in Reference Section**

GRIST-Dev. (2022). DYAMOND winter of GRIST nonhydrostatic model (A21) [Dataset]. Zenodo. 10.5281/ZENODO.6497071.

**Crossref Metadata**


{"key": "e_1_2_7_7_1","doi-asserted-by": "publisher","unstructured": "GRIST‐Dev. (2022).DYAMOND winter of GRIST nonhydrostatic model (A21)[Dataset].Zenodo.10.5281/ZENODO.6497071.","DOI": "10.5281/ZENODO.6497071"},


### Techniques for Identifying a Data or Software Citation to Properly Tag it in the Reference Section Metadata

Citations for data or software can be difficult to discern from journal citations. We offer these techniques for your consideration.

**Request Authors to include Bracketed Descriptions in Data and Software Citations:** As defined by the Publication Manual of the American Psychological Association, 7th Edition, bracketed descriptions “help identify works outside the peer-reviewed academic literature (i.e., works other than articles, books, reports, etc.), provide a description of the work in square brackets after the title and before the period.”

Benefit: Easier identification of data or software citations in the Reference Section. Bracketed descriptions can be added to any flexible journal style guide.

Challenge: At this time there is no broadly accepted standard list of terms and adding a bracketed description for data and software citations adds to the burden on authors and likely will not be consistent.

**Request Authors to include Availability Statement as a Guide to Reviewing the Reference Section for Data and Software Citations**.

Benefit: A complete Availability Statement that has been validated through journal staff and/or peer review can be used to determine which citations in the Reference Section are data or software.

Challenge: Staff, reviewers, and authors need clear guidance on what should be included in the Availability Statement and examples of data and software citations. The publication process should include steps to review and provide guidance to authors on completing or improving the Availability Statement. See the Availability Statement Recommendations Section for a list of elements to include.

**Use Content Negotiation on machine-actionable metadata from the repository landing page**.

Benefit: Crossref and DataCite provide a tool that validates Digital Object Identifiers (https://citation.crosscite.org/docs.html) and returns information that includes the “type” of content registered, such as “data set” or “software.” Content Negotiation often also works with references to URLs, especially when requesting Schema.org (“application/ld + json”) metadata. Many repositories also have machine-actionable metadata in Schema.org format on their landing page, which makes it a universal way to request metadata and type (data/software/creative work).

Challenge: Often, content negotiation only works for DOIs registered with Crossref or DataCite. Additionally, for generalist repositories, the “type” information might not be accurate. Researchers depositing their data without support from a Data Manager will usually select the default type, misidentifying the files. The metadata format of Crossref and DataCite is different, so an implementation for both formats is required. It may also be advisable to request Schema.org metadata.

## Conclusion

Publishers today have a responsibility to the research community, to establish and provide digital connections between the published article and the software and datasets that support that article. This is critical for research transparency, integrity, and reproducibility in today’s data- and software-intensive science enterprise. Scientific communications that do not provide these connections can no longer be considered fully robust.

In implementing required changes to support these responsibilities many journals do not yet have policies and practices strong enough to ensure the necessary dataset and software citations are included in the references. Further, if such citations are included, the journal guidance has heretofore often been murky on how the machine-actionable citations should be digitally formatted for downstream services to provide automated attribution and credit.

This article provides the much-needed guidance to help journals review their production processes and work with their authors, editors, reviewers, staff, and production teams to ensure high-quality dataset and software citations that support automated attribution.

The authors of this article are members of the FORCE11 Journals Task force representing many society, institution, and commercial publishers world-wide. They encourage their peers to adopt these practices and help enable proper machine-actionable dataset and software citations.

Journals should use the Checklist of Best Practices for Journal Publishers located in the results section, to start their journey of improving machine-actionable dataset and software citations.

We strongly encourage our colleagues in academic publishing to adopt these practices. Colleagues with questions, or wishing to share success stories, should contact the lead author.

### Software and data citation resources

This software and data citation resource list includes discipline-agnostic, community-developed and vetted guidance for scholarly publishers, scientific repositories, authors, software developers, and researchers. For a list of relevant software citation resources, see^5,22–24^, and relevant data citation resources, see^2,3^

### Availability statement recommended elements

This text is adapted from AGU’s Data and Software Availability and Citation Checklist (available at: https://data.agu.org/resources/availability-citation-checklist-for-authors).

1. Description of the Type(s) of data and/or software - [Required] Examples:
  - Data - The facilities of IRIS Data Services were used for access to waveforms and related metadata from the International Geodynamics and Earth Tide Service (Network Of Superconducting Gravimeters, 1997)
  - Software - Figures were made with Matplotlib version 3.2.1 (Caswell *et al*., 2020; Hunter, 2007)

2. Repository Name(s) where the data/software are deposited - [Best Practice]
3. URL/link to the data/software, preferably Persistent Identifier (e.g., DOI) and resolves - [Required] Examples:
  - Software - 10.5281/zenodo.3714460
  - Data - 10.7283/633E-1497

4. Access Conditions - [Best Practice] Examples:
  - Registration/fee required
  - Database where certain functionality, selections, or query need to be made. Provide the details.
5. English Translation - [Required] Examples:
  - Site includes translation functionality
  - Translation available via browser plug-in
  - Author guides the readers/makes translation available
6. Licensing - [Best Practice] Examples:
  - Software - MIT License (others)
  - Data - CC-BY (others)
7. In-text Citation where possible - [Best Practice] Examples:
  - Software - Figures were made with Matplotlib version 3.2.1 (Caswell *et al*., 2020; Hunter, 2007), available under the Matplotlib license at https://matplotlib.org/.
  - Data - Data from the KNMI archive with Federation of Digital Seismograph Networks (FDSN) network identifiers NL (KNMI, 1993) and NA (KNMI, 2006) were used in the creation of this manuscript.
8. If software, also include
  - Version (e.g., Version 3.2.1) - [Best Practice]
  - Link to publicly accessible Development Platform (e.g., GitHub) - [Best Practice] Examples:
    i. Part of the software (version 1.0.0) associated with this manuscript for the calculation and storage of PSDs is licensed under MIT and published on GitHub https://github.com/Jollyfant/psd-module/ (Jollyfant, 2021).
  - Author, Project Name(s) instead of username(s) (e.g., username123)
  - Additional Context/Description beyond acronym or code name (e.g., Longhorn pipeline scripts for reducing data vs Longhorn)

### Ethics declarations

This work was conducted as part of the FORCE11 Software Citation Implementation Working Group’s Journal Task Force. We follow the FORCE11 Code of Conduct, https://force11.org/info/code-of-conduct/.

## Data Availability

This research did not use or result in a scientific dataset.

## Code availability

This research did not use or result in software.
